# Lamivudine therapy for chronic hepatitis B in children: a meta-analysis

**DOI:** 10.1186/s12985-019-1193-x

**Published:** 2019-07-04

**Authors:** Aoran Luo, Xiaoyan Jiang, Hong Ren

**Affiliations:** Key Laboratory of Molecular Biology for Infectious Diseases (Ministry of Education), Department of Infectious Diseases, Institute for Viral Hepatitis, The Second Affiliated Hospital, Chongqing Medical University, 1 Yixueyuan Road, Chongqing, 400010 People’s Republic of China

**Keywords:** Lamivudine, Chronic hepatitis B, Children

## Abstract

**Background:**

Currently, there is no consensus on the effects and safety of lamivudine therapy for chronic hepatitis B (CHB) in children.

**Method:**

Both English and Chinese databases were searched comprehensively. An odds ratio (OR) and a standard mean difference (SMD) were used to assess the effects and safety of lamivudine therapy for CHB in children.

**Results:**

Thirteen eligible studies were included in our analysis. The rates of Hepatitis B virus (HBV) response, biochemical response, hepatitis B e antigen (HBeAg) loss, HBeAg seroconversion, and hepatitis B surface antigen (HBsAg) loss were significantly higher in the lamivudine (LAM) therapy group than in the control group. The changes in children’s weight and height were similar between the two groups.

**Conclusions:**

LAM therapy was efficacious for CHB in children. Additionally, it had no side effect on children’s height and weight.

**Electronic supplementary material:**

The online version of this article (10.1186/s12985-019-1193-x) contains supplementary material, which is available to authorized users.

## Background

Chronic hepatitis B (CHB) virus infection is a serious global health problem with approximately 3.6% of the world’s population suffering from chronic hepatitis B infection [1]. Hepatitis B virus (HBV) is transmitted vertically (from mother to child at birth) and horizontally (from person to person) [2, 3]. Perinatal infection of infants is from HBeAg-positive mothers, which is common in southeast Asia, while HBeAg-positive mothers are not common in eastern Europe, Africa, and the Mediterranean basin [4, 5]. In these areas, children are infected by close contact with HBsAg-positive individuals during early childhood, and more importantly, up to 90% of perinatal infections become chronic diseases [6]. The positive rate of hepatitis B surface antigen in children varies by region. It is reported that the highest rate observed is 8.8% in Uganda [2].

The current treatments for children with CHB are interferon-α and four nucleoside analogs: IFN-α initiated in children 12 months and older; LAM for children 3 years and older; adefovir and tenofovir initiated in children 12 years and older; and entecavir initiated in children 16 years of age [7]. LAM is the first and primary antiviral drug currently officially approved for children with CHB under 12 years old. In addition, LAM is also less expensive than the other three NAs. Therefore, LAM is the first choice, particularly for children in developing countries, despite a low genetic barrier against the increase in resistance.

Because there are too few studies published in English investigating lamivudine treatment in children with hepatitis B infection, a recent meta-analysis of work examining the management of CHB in children only included one relevant study [8]. Moreover, recent emerging clinical studies have not been consistent. To synthesize research regarding lamivudine treatment of hepatitis B in children around the world, not only the English databases but also the Chinese databases were searched for the present meta-analysis.

## Materials and methods

### Search strategy

Relevant studies were found by searching the English-language databases (EMBASE, PubMed, Web of Science, the Cochrane Library, and ClinicalTrials.gov) and Chinese-language databases [China National Knowledge Infrastructure (CNKI) and the Chinese BioMedical Literature Database (CBM)], using the following strategy:(((newborn* or neonat* or infant* or child* or adolescent* or paediatric* or pediatric*)) AND (lamivudine OR ‘2,3 dideoxy 3 thiacytidine’ OR ‘3TC’ OR epivir OR ‘lamivudine, (2S-cis)-isomer’ OR ‘BCH 189’ OR ‘GR109714X’)) AND (HBV OR hepatitis B). We included all cohort and randomized controlled trials (RCTs). The search was conducted in December 2018. The reference lists of all retrieved review articles were manually searched for potentially relevant articles missed by the intelligent retrieval system.

### Selection criteria

The inclusive clinical trials had to fulfill the following criteria: (1) Study design: RCTs, with retrospective and prospective cohort study designs (each group sample size > 10); (2) Subjects: children or adolescents under the age of 18 with chronic hepatitis B; (3) Treatment strategy: including a LAM (100 mg/day) monotherapy group and a placebo or general treatment group as a control group; (4) Outcome: including virological responses, such as rates of HBV response, HBeAg loss and HBeAg seroconversion, or biochemical responses, such as rates of Alanine Transaminase (ALT) and Aspartate Transferase (AST) normalization. The exclusion criteria were as follows: (1) duplicated data; (2) coinfection with hepatitis A, C, D, or E viruses or human immunodeficiency virus; (3) Wilson’s disease, autoimmune hepatitis, primary biliary cirrhosis, hepatocellular carcinoma, etc.; (4) any report without sufficient information.

### Outcome measures

End-points were defined before the initiation of the study. The primary efficacy end-point was the rate of HBV virological response that was by definition as the proportion of patients with undetectable serum HBV-DNA after treatment. The secondary efficacy end-points were as follows: HBeAg conversion rate, HBeAg loss rate; HBsAg loss rate; and biochemical response, defined as the normalization of ALT and AST. Safety end-points were height and weight changes after treatment.

### Study quality assessment

The revised Jadad quality scale was used to evaluate the quality of all 8 RCTs included in the meta-analysis by examining the description of the randomization and blinding methods and the description of deviations and drop-outs. Out of the 8 RCTs, only one received a Jadad score of 6, and the Jadad scores were 3 for four studies and 2 for the remaining three studies. All 8 studies claimed to be RCTs, while only one study reported the randomization method. Only one of the studies was blinded. Four studies described study withdrawals and dropouts in detail. The Newcastle-Ottawa Scale (NOS) was used to evaluate the five included cohort studies based on several standards, including the selection of cohorts, comparability of cohorts, and assessment of the outcomes. Of the five cohort studies, only one received a NOS score of seven, and the four other studies received a score of six.

### Data extraction

Using the same data collection table, the data for each included study were extracted independently and in duplicate by the two authors (Aoran Luo, and Xiaoyan Jiang). The data were extracted for (1) study characteristics (author, year of publication, geographic locale, study design, regimen, duration of follow up and sample size); (2) patient demographics (age, sex) and baseline characteristics (HBeAg-positive percentage, alanine aminotransferase levels, and serum HBV DNA levels); and (3) study outcomes after treatment. Any disagreement between the reviewers was resolved by a third party (Hong Ren).

### Statistical analysis

All statistical analyses were carried out with Stata (version 12.0). For each included study, the dichotomous results were presented using the odds ratio (OR) with a 95% confidence interval (95% CI), while the continuous results were presented using a standardized mean difference (SMD) with a 95% confidence interval (95% CI). The statistical heterogeneity was assessed by using chi-square and I-square (I^2^) tests with the significance level set at *p* < 0.1. If significant heterogeneity was not present in the data, a fixed-effects model was adopted for analysis; otherwise, a random-effects model was adopted. In addition, a Galbraith plot and a sensitivity analysis were employed to explore sources of heterogeneity. Finally, funnel plots were constructed for eligible outcomes, together with Egger’s tests to examine the possible publication bias [9]. All *P* values were two-sided. Apart from Cochran’s Q-test, the significance level was set at *P* < 0.05.

## Results

### Search results and study characteristics

**Fig. 1:**
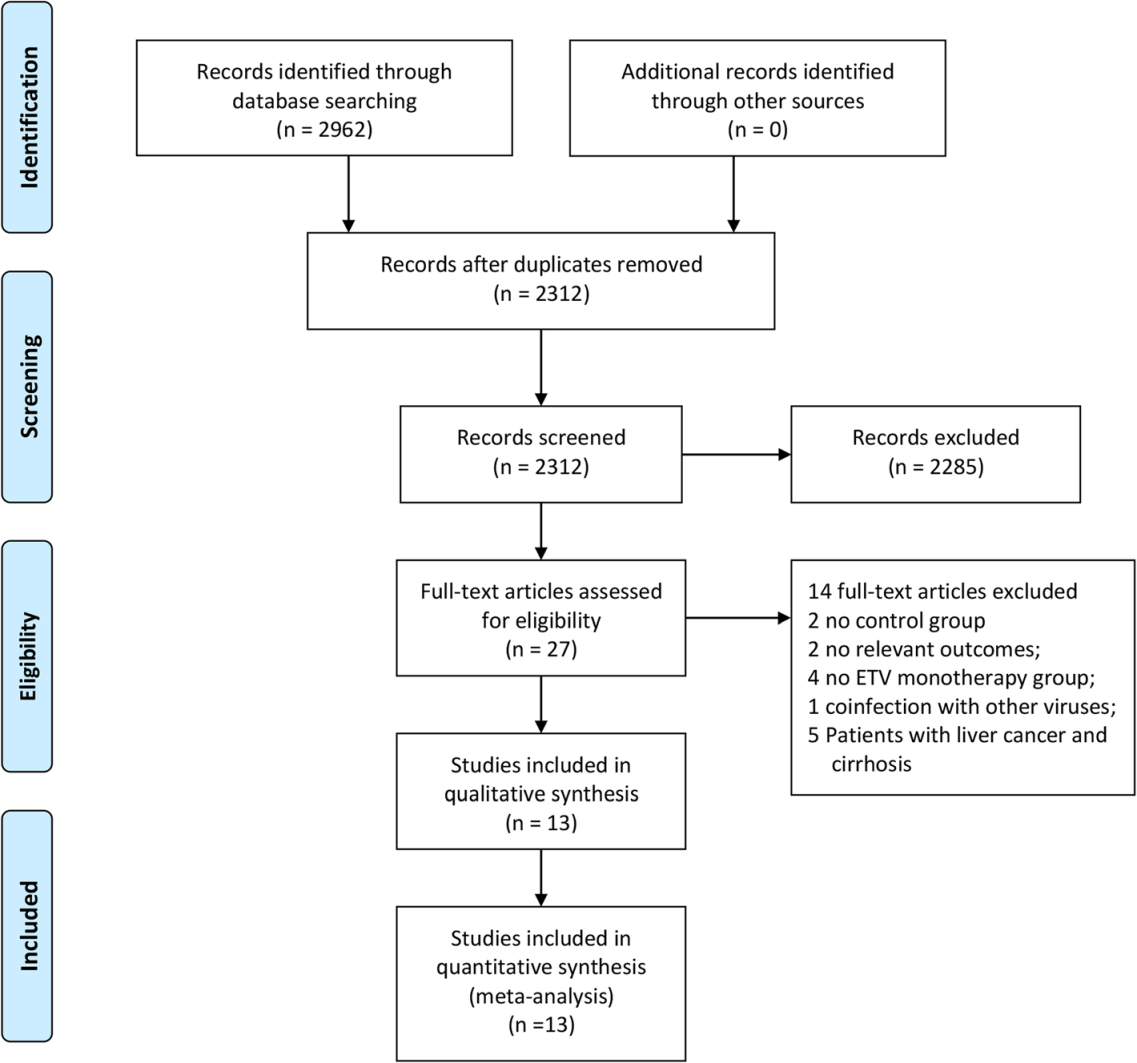
Study selection process

A total of 2962 studies were identified by using the strategy described above. A total of 650 duplicates were excluded. A total of 2285 records were excluded after scanning titles and abstracts. Finally, 8 randomized controlled trials and 5 cohorts were included in the meta-analysis, which involved a total of 1556 patients. Figure 1 shows the study selection process.

The basic characteristics of the 13 studies and the included patients are listed in Tables 1 and 2. Eleven of the included studies were from China, and the other 2 studies were from European and American countries. The included studies were published between 2002 and 2014. The sample size for each study ranged from 58 to 218. The average age ranged from 8 to 14 years old. The duration of follow-up ranged from 24 to 208 weeks. The percentage of males ranged from 46 to 74%. Only three of the 13 articles reported follow-up after the end of treatment [10–12]. In two of the reports, the follow-up time was 24 weeks and 48 weeks after the end of treatment respectively [10, 12]. and another study only described the follow-up and did not indicate how long to follow up after treatment [11].

**Table 1:** Characteristics of the included trials in this meta-analysis

Author	Year	Geographic Locale	Study Design	Regimen	Sample size	Duration, weeks
Jonas	2002	North America, South America, and Europe	RCT	3 mg/kg· d	286	52
Figlerowicz	2005	Poznan	cohort	3 mg/kg· d	152	48
Zhang	2011	China, Henan province	RCT	3 mg/kg· d	100	24
Luo	2006	China, Hunan province	RCT	3 mg/kg· d	58	72
Wang	2014	China, Hubei province	cohort	3 mg/kg· d	80	52
He	2007	China, Sichuan province	RCT	3 mg/kg· d	193	52
Feng	2011	China, Shanxi province	RCT	3 mg/kg· d	113	60
Wang	2012	China, Jiangxi province	cohort	3 mg/kg· d	218	208
Liu	2006	China, Jilin province	cohort	3 mg/kg· d	70	52
Xu	2004	China, Guangdong province	RCT	3 mg/kg· d	63	96
Wang	2013	China, Jiangxi province	RCT	3 mg/kg· d	80	72
Gao	2002	China, Shandong province	RCT	3 mg/kg· d	80	52
Yang	2010	China, Jiangxi province	cohort	3 mg/kg· d	63	48

Sample size and duration were expressed in mean

**Table 2:** Characteristics of the included patients in this meta-analysis

Author	Year	Age	Sex (male%)	HBV DNA (log10)	HBeAg (+), %	ALT, U/L
Jonas	2002	8.67	64	6442.2^a^	NR	286
Figlerowicz	2005	11	66	NR	100	72
Zhang	2011	11.59	56	NR	100	181
Luo	2006	7.9	58	NR	NR	NR
Wang	2014	NR	NR	NR	NR	NR
He	2007	11^a^	58	5.58	100	100
Feng	2011	7.5	51	NR	100	150
Wang	2012	11.2	52	6.54	100	248
Liu	2006	12	50	NR	100	NR
Xu	2004	5.6	46	NR	100	99
Wang	2013	10.24	NR	6.79	100	202
Gao	2002	13.5	74	5203.2^a^	80	224
Yang	2010	10.16	NR	6.77	95	202

HBV DNA, HBeAg, ALT, and CD4^+^T cell were all expressed in mean. NR: not record. ^a^meq/ml

### Comparison of rates of HBV response in the LAM therapy group and control group

**Fig. 2:**
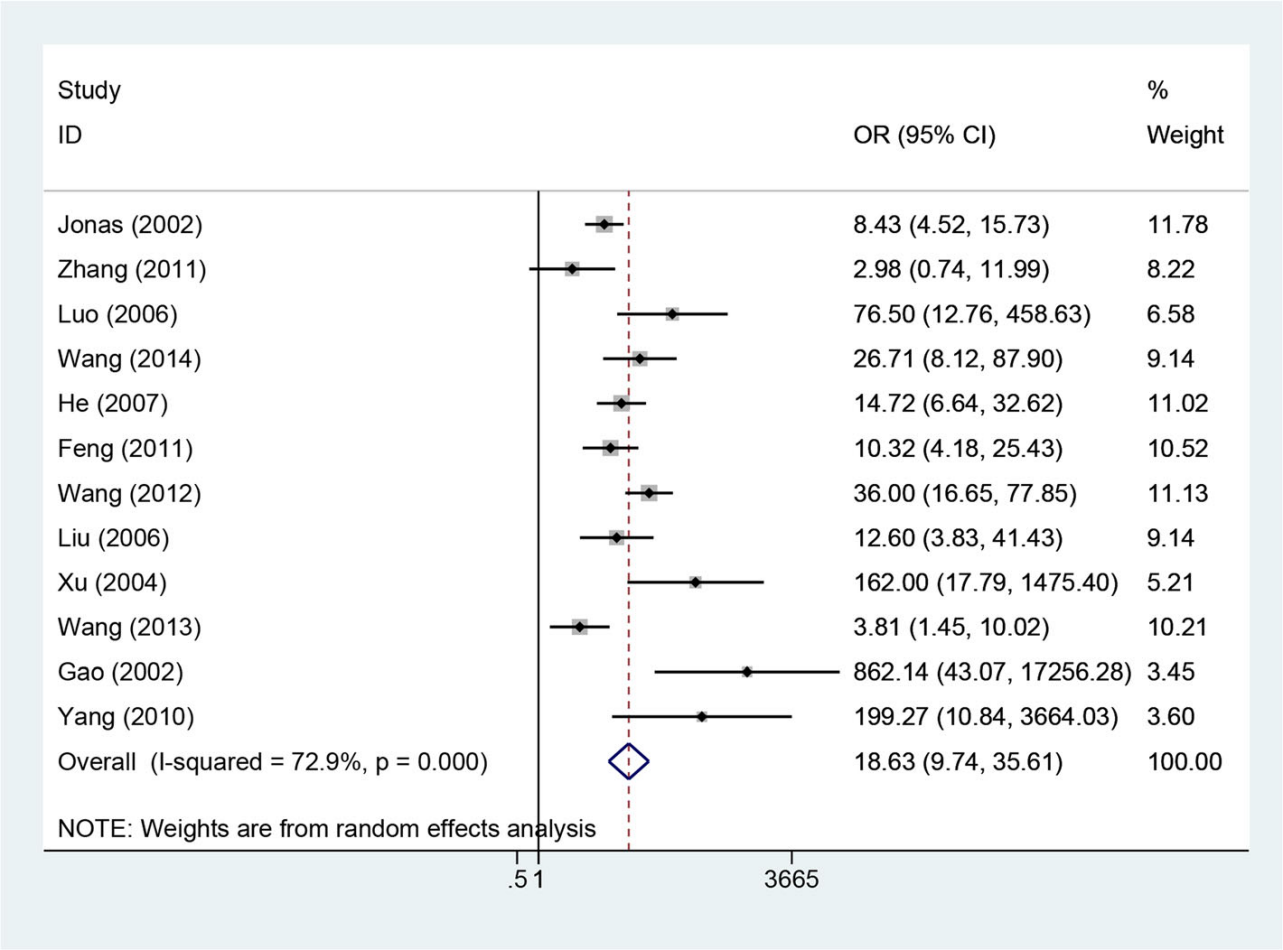
Effect of LAM vs. control group on HBV virologic response

Twelve included studies, involving 1402 patients, reported undetectable rates of HBV DNA [11–22]. As the heterogeneity among these studies was significant (*P* = 0.0001, I^2^ = 72.9%), the random-effects method was applied to calculate the overall effects. The rate of HBV response was higher in the LAM group than in the control group (OR = 18.63, 95% CI: 9.75–35.61, P = 0.0001; Fig. 2). Subgroup analysis according to study design showed that the rate of undetectable HBV DNA was higher in LAM groups than in control groups for both RCT and cohort studies. Based on a symmetrical funnel plot and Egger’s tests (*P* = 0.068), no evidence of publication bias was identified.

### Comparison of liver biochemical parameters in the LAM therapy group and control group

**Fig. 3:**
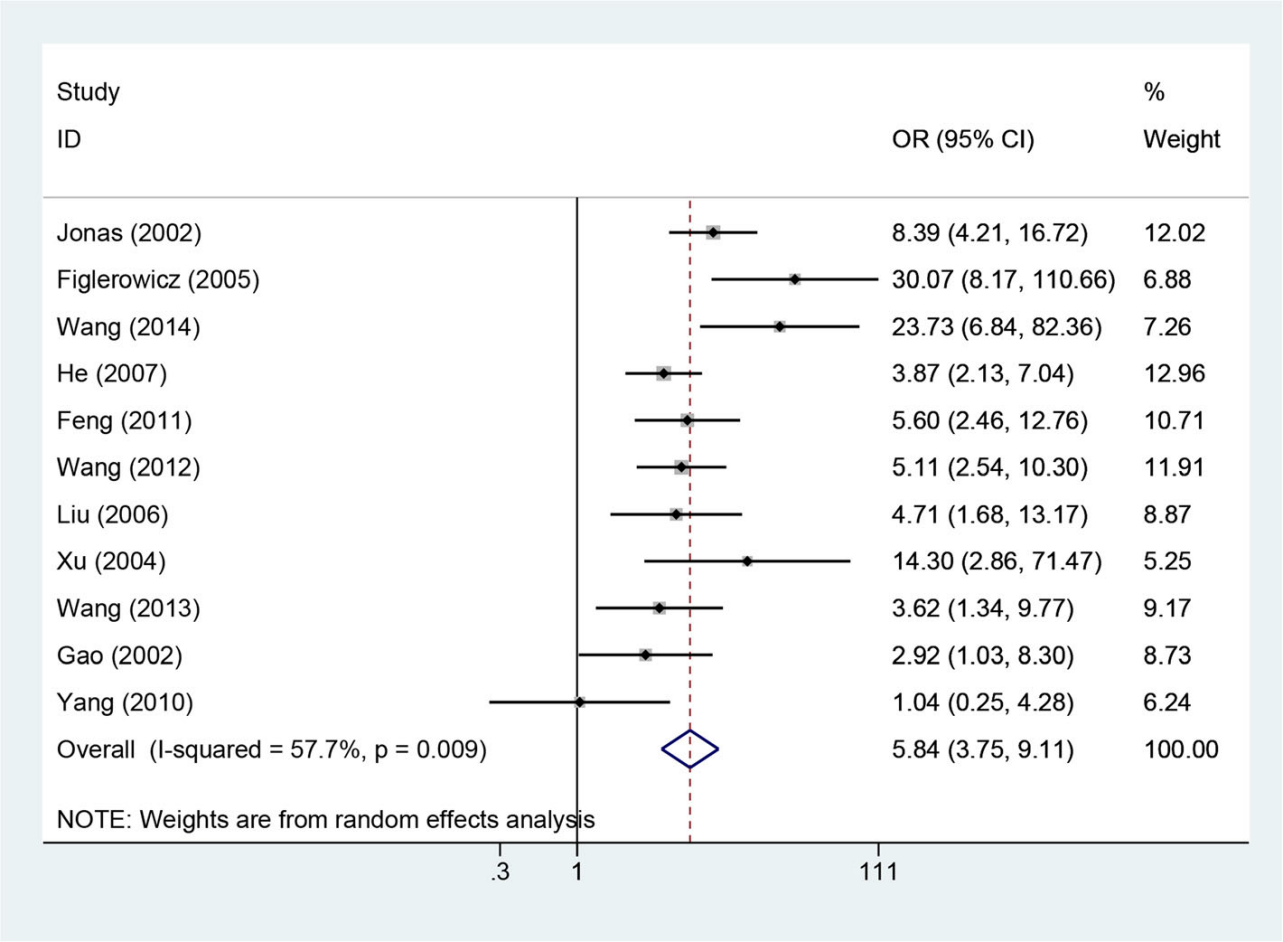
Effect of LAM vs. control group on ALT normalization rate

Eleven included studies involving 1351 patients reported rates of ALT normalization [10–12, 15–22]. The between-study heterogeneity was significant when the 11 studies were pooled into a meta-analysis (*P* = 0.009, I^2^ = 57.7%); thus, the random-effect model was adopted to pool the results. The results suggested that the rate of ALT normalization was higher in the LAM therapy group than in the control group (OR = 5.84, 95% CI: 3.75–9.11, *P* = 0.0001; Fig. 3). Subgroup analysis according to study design suggested that the rate of ALT normalization was higher in LAM groups than in control groups for both RCT and cohort studies. Subgroup analysis by area showed the same result for both European and American countries and China.

Three included studies involving 223 patients reported rates of AST normalization [12, 15, 21]. The heterogeneity was significant when the 3 studies were pooled into a meta-analysis (*P* = 0.063, I^2^ = 63.9%); thus, the random-effects model was adopted to pool the results. The meta-analysis showed that the rate of AST normalization was higher in the LAM therapy group than in the control group (OR = 8.46, 95% CI: 2.75–26.01, *P* = 0.0001; Additional file 1: Figure S1). There was no evidence of publication bias according to funnel plot analysis and Egger’s tests (*P* = 0.494).

### Comparison of HBeAg seroconversion rates and HBeAg loss in the LAM therapy group and control group

**Fig. 4:**
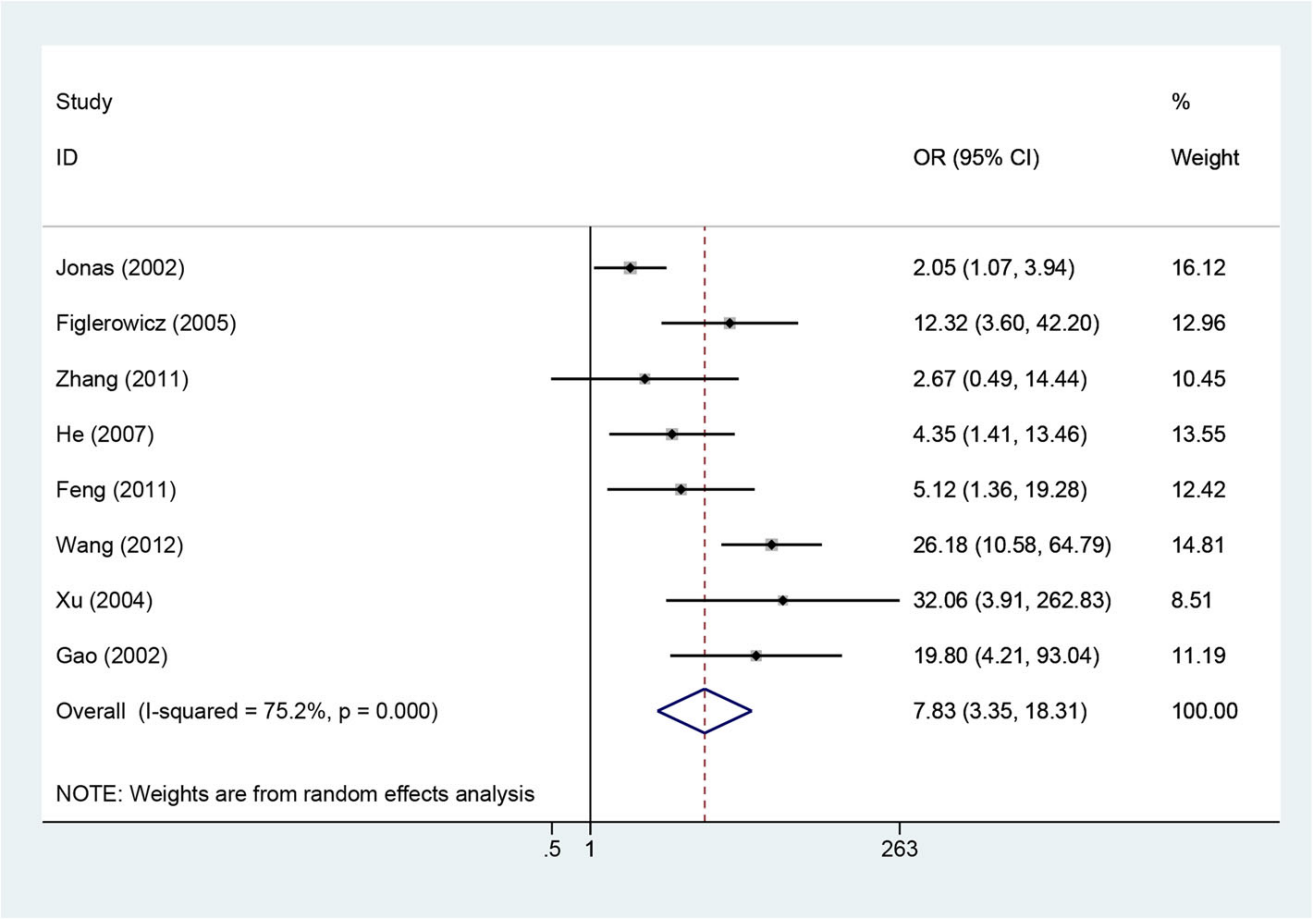
Effect of LAM vs. control group on HBeAg loss rate

**Fig. 5:**
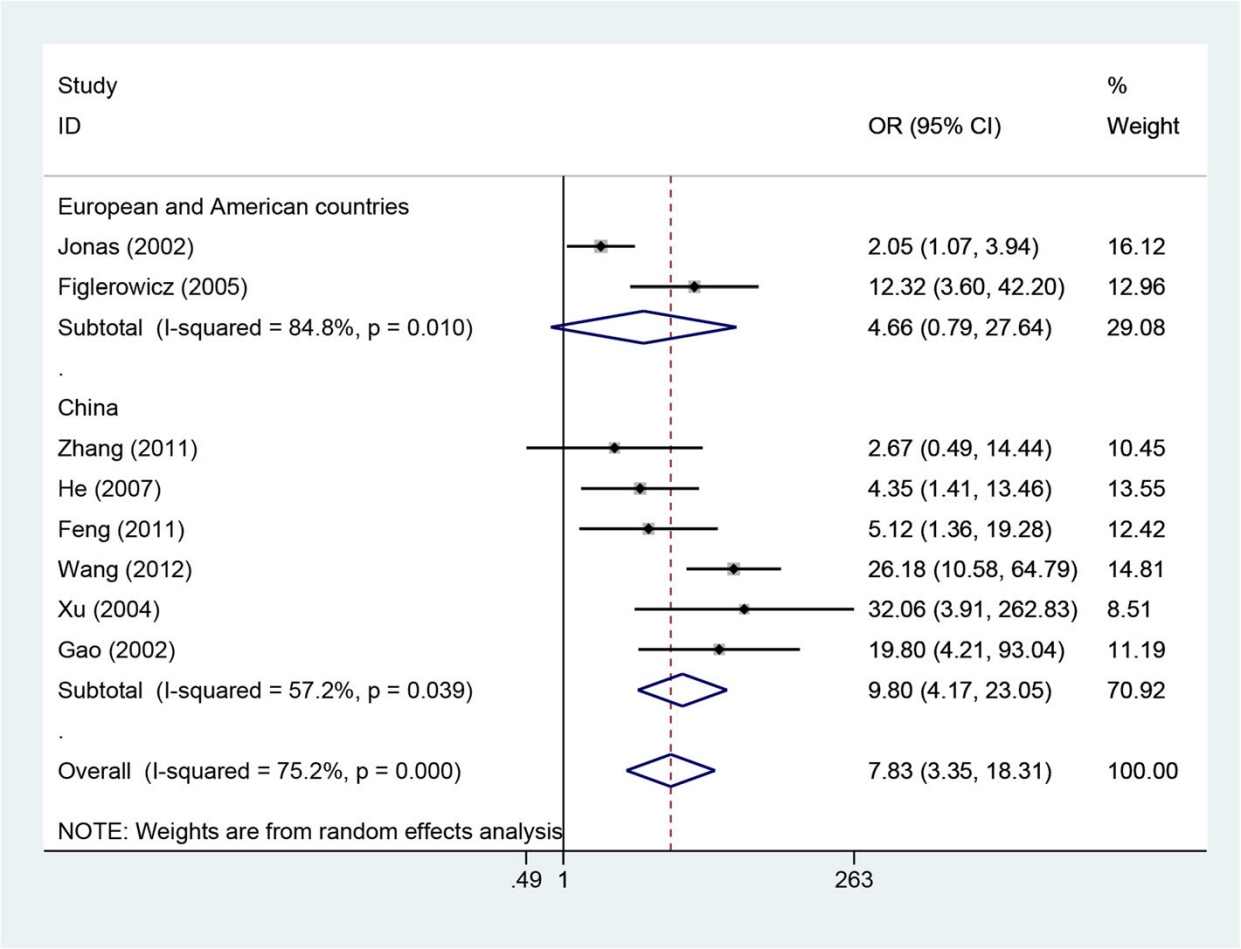
Subgroup analyses by area for HBeAg loss rate

Eight included studies involving 1402 patients reported rates of HBeAg loss [10–13, 16–18, 21]. Because the heterogeneity was significant among these studies (*P* = 0.0001, I^2^ = 75.2%), the random-effects method was applied to calculate the overall effects. The rate of HBeAg loss was higher in the LAM group than in the control group (OR = 7.83, 95% CI: 3.35–18.31, P = 0.0001; Fig. 4). Subgroup analysis according to study design suggested that the rate of HBeAg loss was higher in LAM groups than in control groups for both RCT and cohort studies. Subgroup analysis by area showed that the HBeAg loss rate was similar between the two groups for both European and American countries, but for studies from China, the rate of HBeAg loss was higher in the LAM group than in the control group (OR = 4.66, 95% CI: 0.79–27.64, *P* = 0.010; OR = 9.80, 95% CI: 4.17–23.05, *P* = 0.039; Fig. 5).

**Fig. 6:**
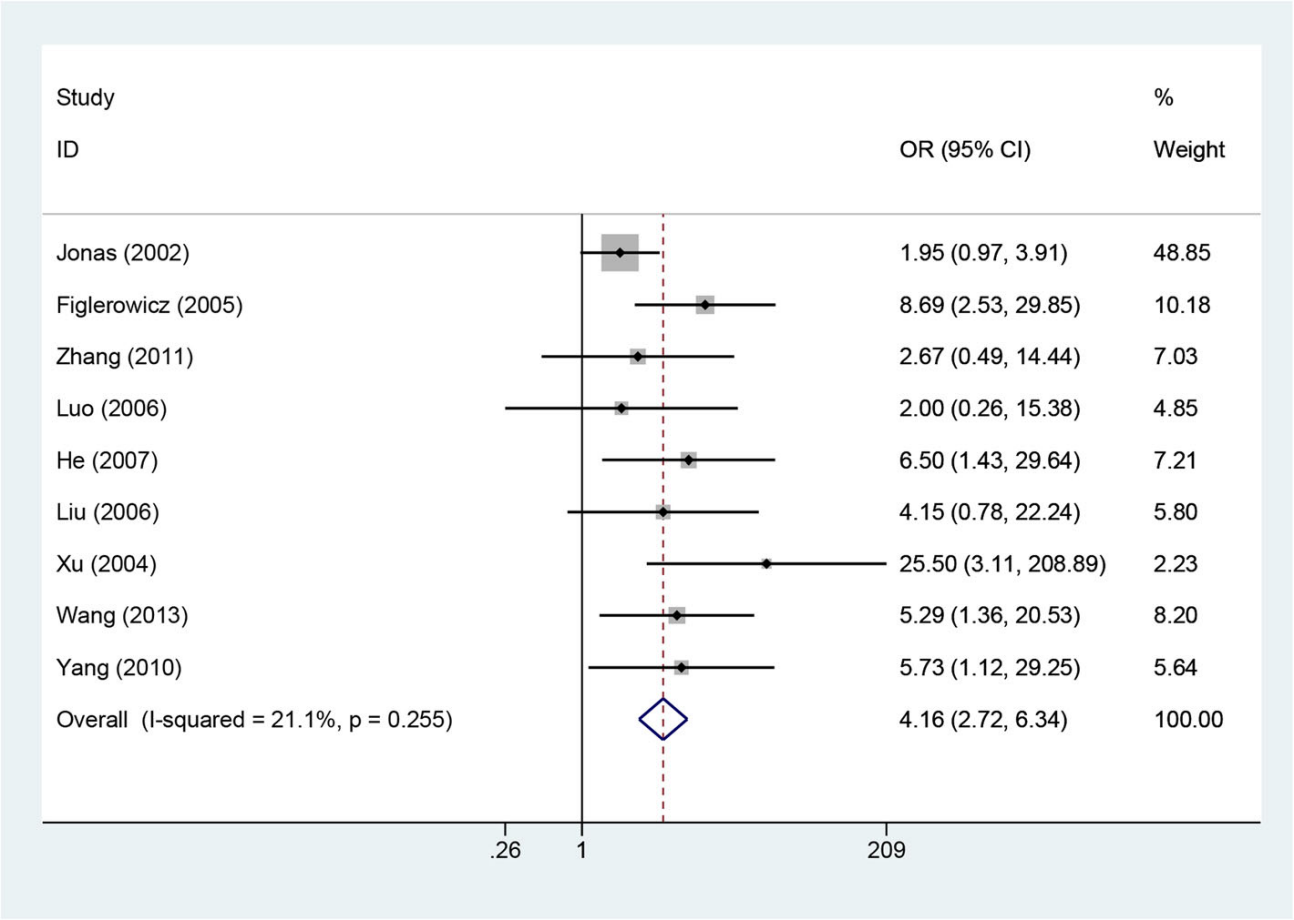
Effect of LAM vs. control group on HBeAg seroconversion rate

Nine included studies involving 1063 patients reported rates of HBeAg seroconversion [10–14, 16, 19, 20, 22]. The heterogeneity was not significant when the 9 studies were pooled into a meta-analysis (*P* = 0.255, I^2^ = 21.1%); thus, the fixed-effects model was assumed to pool the results. The results suggested that the HBeAg seroconversion rate was higher in the LAM therapy group than in the control group (OR = 4.16, 95% CI: 2.72–6.34, *P* = 0.0001; Fig. 6). Subgroup analysis based on study design suggested that the HBeAg seroconversion rate was higher in LAM groups than in control groups for both RCT and cohort studies. Subgroup analysis by area showed the same result for both European and American countries and China.

### Comparison of HBsAg loss rates in the LAM therapy group and control group

Four included studies involving 485 patients reported rates of HBsAg loss [11, 14, 15, 22]. As there was no significant heterogeneity among these studies (*P* = 0.361, I^2^ = 6.4%), the fixed-effects method was applied to combine the overall effects. The HBsAg loss rate was higher in the LAM group than in the control group (OR = 15.30, 95% CI: 5.25–44.56, *P* = 0.0001; Additional file 2: Figure S2).

### Safety comparison in the LAM therapy group and control group

Most studies assess the safety of lamivudine by comparing changes in children’s weight and height. Therefore, we combined these two outcomes as follows. Three included studies involving 334 patients reported height and weight changes [16, 20, 22]. The heterogeneity was not significant when the studies were pooled into a meta-analysis (*P* = 0.813, I^2^ = 0.01%; *P* = 0.999, I^2^ = 0.01%); thus, the fixed-effects model was adopted to pool the two results. The results suggested that both the height and weight gains were similar between the two groups (OR = 0.001, 95% CI: − 0.21–0.22, *P* = 0.967, Additional file 3: Figure S3; OR = 0.06, 95% CI: − 0.16–0.27, *P* = 0.609, Additional file 4: Figure S4).

### Comparison of LAM resistance rates in the LAM therapy group and control group

Four included studies involving 461 patients reported rates of LAM resistance rates [11, 15, 19, 22]. As there was no significant heterogeneity among these studies (*P* = 0.64, I^2^ = 0%), the fixed-effects method was applied to combine the overall effects. The LAM resistance rate was higher in the LAM group than in the control group (OR = 15.46, 95% CI: 3.55–67.25, *P* = 0.0003).

## Discussion

Currently, the first-line medication for hepatitis B patients in children under the age of 12 is mainly interferon and lamivudine. Because interferon is injected subcutaneously, while lamivudine is administered orally, lamivudine is more convenient for the patient and therefore more widely used. However, at present, there is only one large-scale clinical trial of lamivudine, published in the New England Journal of Medicine [10]. Moreover, a recent meta-analysis of studies examining the management of chronic hepatitis B viral infection in children only included this study [8], but related experiments have been published continuously in recent years. It is necessary to conduct a meta-analysis to summarize the worldwide data. The present meta-analysis was performed by carefully reviewing 8 individual RCT studies and 5 cohort studies to compare outcomes related to chronic hepatitis B in children between LAM therapy and control groups. Subgroup analyses were primarily addressed through the study design or by the area in European and American countries and China.

The current meta-analysis demonstrates that lamivudine is effective in reducing the rates of HBV DNA response, HBeAg loss, HBeAg seroconversion and HBsAg loss and improving liver biochemical parameters in children who are infected with HBV. In addition, our results show that lamivudine therapy has no negative impact on children’s height and weight. It was unexcepted that subgroup analysis by area showed that the HBeAg loss rate was similar between the two groups for both European and American countries, but for studies from China, the HBeAg loss rate was higher in the LAM group than in the control group. The following reasons may explain this result: (1) genotype A is prevalent in European and American countries, while genotype B and genotype C are prevalent in China; (2) there are too few relevant studies in European and American countries to achieve statistical significance in differences between the LAM and control groups.

Although lamivudine can effectively inhibit the growth of the virus and improve liver inflammation, the use of lamivudine still has two limitations: (1) pre-existing covalently closed circular DNA in the liver cannot be eliminated by nucleoside analogs, including LAM, so relapse is frequent when therapy is withdrawn; (2) the emergence of resistant strains called YMDD (tyrosine, methionine, aspartate, aspartate) could lead to virological breakthrough followed by biochemical breakthrough. Therefore, LAM should be used carefully in the treatment of hepatitis B in children. Some studies found that the combination of interferon and lamivudine can improve these two aspects of lamivudine defects. Chan et al. found that a lower rate of LAM resistance emerged with a combination therapy of peg-IFN and LAM (21%) compared with LAM monotherapy (40%) [23]. Selimoglu et al. showed the rate of breakthrough to be 23.4% in children treated with IFN-α and LAM combination therapy. Hence, the combination of interferon and lamivudine may be a good choice, especially for children under 12 years of age [24]. Moreover, large prospective randomized trials examining the use of nucleoside analogs with higher genetic resistance barriers (Entecavir and Tenofovir disoproxil) in children with hepatitis B infection have been largely lacking. Otherwise, for patients under 12 years of age, there may be more effective treatment.

Some possible limitations of this study should be considered before generalizing our findings. First, 8 randomized controlled trials and 5 cohort studies were included, so not all of the included studies were randomized controlled trials. Second, of the 1556 patients included, 438 were from European and American countries, and the remaining 1118 were from China. Third, due to the limited number of studies included, information on individual patients was not detailed enough to evaluate the treatment effects in the different subgroups. Fourth, the durations of lamivudine use were different, which probably impacted the treatment effect of LAM therapy. Fifth, as the revised Jadad quality scale showed, the quality of the randomized controlled trials included here was not very high. Despite these limitations, the present meta-analysis summarized the newest worldwide data on lamivudine treatment for children. However, significant results were found primarily for Chinese but not for other populations. Large and well-designed studies in a variety of populations worldwide are needed to better generalize the present results.

## Conclusions

LAM therapy was efficacious for CHB in children. Additionally, it had no side effect on children’s height and weight.

## Additional files

Additional file 1: **Figure S1.** Effect of LAM vs. control group on AST normalization rate. (TIF 440 kb)

Additional file 2: **Figure S2.** Effect of LAM vs. control group on HBsAg Loss rate. (TIF 468 kb)

Additional file 3: **Figure S3.** Effect of LAM vs. control group on height gains of children rate. (TIF 399 kb)

Additional file 4: **Figure S4.** Effect of LAM vs. control group on weight gains of children rate. (TIF 406 kb)

## Data Availability

All relevant data are within the paper and its Supporting Information files.
